# Early Seizure Detection Based on Cardiac Autonomic Regulation Dynamics

**DOI:** 10.3389/fphys.2017.00765

**Published:** 2017-10-05

**Authors:** Jonatas Pavei, Renan G. Heinzen, Barbora Novakova, Roger Walz, Andrey J. Serra, Markus Reuber, Athi Ponnusamy, Jefferson L. B. Marques

**Affiliations:** ^1^Department of Electrical and Electronic Engineering, Institute of Biomedical Engineering, Federal University of Santa Catarina, Florianópolis, Brazil; ^2^Department of Neurology and Clinical Neurophysiology, Royal Hallamshire Hospital, Sheffield Teaching Hospitals NHS Foundation Trust, University of Sheffield, Sheffield, United Kingdom; ^3^Neurology Unit, Department of Clinical Medicine, Federal University of Santa Catarina, Florianópolis, Brazil; ^4^Biophotonic Laboratory, Nove de Julho University, São Paulo, Brazil

**Keywords:** electrocardiogram, heart rate variability, epilepsy, epileptic seizure prediction, support vector machines

## Abstract

Epilepsy is a neurological disorder that causes changes in the autonomic nervous system. Heart rate variability (HRV) reflects the regulation of cardiac activity and autonomic nervous system tone. The early detection of epileptic seizures could foster the use of new treatment approaches. This study presents a new methodology for the prediction of epileptic seizures using HRV signals. Eigendecomposition of HRV parameter covariance matrices was used to create an input for a support vector machine (SVM)-based classifier. We analyzed clinical data from 12 patients (9 female; 3 male; age 34.5 ± 7.5 years), involving 34 seizures and a total of 55.2 h of interictal electrocardiogram (ECG) recordings. Data from 123.6 h of ECG recordings from healthy subjects were used to test false positive rate per hour (FP/h) in a completely independent data set. Our methodological approach allowed the detection of impending seizures from 5 min to just before the onset of a clinical/electrical seizure with a sensitivity of 94.1%. The FP rate was 0.49 h^−1^ in the recordings from patients with epilepsy and 0.19 h^−1^ in the recordings from healthy subjects. Our results suggest that it is feasible to use the dynamics of HRV parameters for the early detection and, potentially, the prediction of epileptic seizures.

## 1. Introduction

Epilepsy is a chronic disorder that has a significant impact on patients' quality of life and health care budgets. The prevalence of epilepsy has been reported to range from 0.5 to 2% in the general population (Nunes et al., 2011). It is characterized by sudden recurrent and transient disturbances of perception or behavior resulting from the excessive synchronization of cortical neuronal networks due to abnormal bursts of electrical discharge in the brain (Tzallas et al., 2012). One of the most disabling aspects of the disorder is the unpredictability of the seizures.

Although a wide range of drugs and surgical treatments are available, seizures remain uncontrolled in over 25% of patients (Valderrama et al., 2012). Most epileptic seizures are self-limiting but they occasionally develop into a potentially life-threatening, more persistent condition (status epilepticus). Although most seizures, including generalized tonic clonic seizures (GTCS), do not cause lasting damage to the brain, they are associated with a small risk of death due to cardiac or respiratory complications sudden unexpected death in epilepsy (SUDEP). The SUDEP risk is particularly high in individuals who sleep on their own and have GTCS during sleep (Lamberts et al., 2012). Studies have shown that individuals with epilepsy often remain unaware of their own seizures, especially the milder seizures and seizures associated with sleep (Hoppe et al., 2007). Thus, there is a great interest in the development of reliable tools for early seizure detection, and potentially for seizure prediction, in order to allow acute intervention or, at least, to give patients an opportunity to prepare themselves for a seizure (Carney et al., 2011).

Most techniques for the detection and prediction of epileptic seizures involve linear and non-linear processing of electroencephalographic (EEG) signals, which reflect the electrical activity in the brain (Acharya et al., 2012; Rana et al., 2012; Duque-Munoz et al., 2014; Hassan et al., 2016; Li et al., 2016). Some studies have achieved excellent results, with 100% accuracy for seizure detection (Alam and Bhuiyan, 2013). In terms of prediction, one of the best techniques achieved a sensitivity of 97.5% and a false positive rate of 0.27 h^−1^ (Park et al., 2011).

Previous studies have indicated that the analysis of autonomic nervous system (ANS) activity may help to identify epileptic seizures. Information on ANS activity can be obtained through heart rate variability (HRV) analyses. HRV analyses are based on the measurement of the time intervals between successive QRS complexes, which reflect the regulation of the heart rate by the ANS via its sympathetic and parasympathetic control mechanisms (Ponnusamy et al., 2012). This means that HRV analyses can be used to provide indirect clues about nervous system activity.

Therefore, electrocardiographic (ECG) signals have been used for seizure detection and prediction, based on ECG signals associated with established seizures. In 2009, one study reported that this technique had a sensitivity of 85.7% and a specificity of 84.6% (Malarvili and Mesbah, 2009). Subsequently, Varon et al. (2014) used ECG signals to achieve a positive predictive value (PPV) of 86.2% and a sensitivity of 100% for partial seizures, and a PPV of 84.3% and a sensitivity of 93.1% for generalized seizures. More recently, researchers described a multivariate statistical process for epileptic seizure prediction with a sensitivity of 91% and false positive rate of 0.7 h^−1^ (Fujiwara et al., 2016).

Although these studies demonstrate that established seizures can be reliably detected using ECG signals, it remains uncertain how well HRV-derived parameters could work for the early detection or prediction of seizures. As differences between interictal and preictal HRV parameters have been noted in several studies (Ponnusamy et al., 2011, 2012; Behbahani et al., 2013; Pavei et al., 2014), the utility of these differences was investigated in the present study.

EEG-based seizure detection algorithms depend on the detection of specific ictal EEG patterns. Ictal EEG patterns differ between epileptic seizure types. Seizures are detected more reliably by systems involving a larger number of electrodes attached to the scalp or intracranial electrodes which would need to be surgically implanted (and would therefore be associated with risks, such as bleeding or infection). ECG signals are much more readily accessible and can be picked up reliably using non-invasive means (such as wristbands or stick-on electrodes). The ECG signal is also much less complex than EEG signals and can be interpreted with more limited computational resources. Heart rate changes associated with seizures are more generic and have been well-studied previously (Eggleston et al., 2014). In-loop ECG recording devices based on heart rate changes have already been in routine clinical use for several years in Vagus Nerve Stimulators. Therefore, ECG-based seizure detection is currently more promising that EEG-based approaches (Boon et al., 2015).

The aim of this study was to explore the feasibility of using a method for the early detection or forecasting of epileptic seizures based on a set of HRV parameters, in which the principal components of the HRV parameter covariance matrix were used as inputs for a support vector machine (SVM). The feasibility assessment was carried out with ECG recordings of 34 temporal lobe epileptic seizures (TLE), 55.2 h of interictal recordings from patients with TLE, and 123.6 h of recordings from healthy subjects.

## 2. Materials and methods

### 2.1. Subjects

To address the objective of this study (i.e., establishing the feasibility of seizure detection/forecasting) and to minimize physiological variability, we focused exclusively on ECG recordings capturing occurrences of focal seizures from patient with temporal lobe epilepsy. Some patients had secondary generalized seizures, but ECG recordings from this phase of the seizures are not included as we analyze HRV during the interictal and preictal phase of the focal seizures for early seizure detection.

We collected ECG data during clinical video-electroencephalographic (V-EEG) recordings of seizures in 12 patients (9 female; 3 male; age 34.5 ± 7.5 years) with temporal lobe epilepsy (see Table 1 for clinical details). All the ECG recordings were recorded whilst the patients were hospitalized for inpatient V-EEG monitoring at the Santa Catarina Epilepsy Center (CEPESC), which is a regional referral center for patients with refractory epilepsy in the state of Santa Catarina in southern Brazil.

**Table 1:** Summary data for the subjects studied.

**Patient**	**Recording type**	**Gender**	**Age**	**Localization of**	**Type of**	**Drugs**
				**seizure onset**	**seizure**	
P1	Scalp	F	43	LTL	CP	CBZ 400 mg, RIS 1 mg
P2	Scalp	F	36	LTL	CP	CBZ 200 mg, CLB 10 mg
P3	Scalp	F	40	RTL	CP	CBZ 200 mg, PB 100 mg
P4	Scalp	F	31	LTL	CP	CBZ 200 mg, CLZ 2 mg
P5	Scalp	F	32	LTL	CP	CBZ 200 mg, PB 100 mg
P6	Scalp	M	51	LTL	CP	CBZ 200 mg, VPA 500 mg
P7	Scalp	F	29	RTL	CP	CBZ 200 mg, CLB 20 mg
P8	Intracranial EEG	F	30	RTL	CP	CBZ 400 mg
P9	Intracranial EEG	M	33	LTL	CP	CBZ 200 mg, CLB 20 mg
P10	Intracranial EEG	F	48	LTL	CP	OCBZ 600 mg, PB 100 mg
P11	Intracranial EEG	F	42	LTL	CP	VPA 500 mg, PB 100 mg
P12	Intracranial EEG	M	30	RTL	CP	CBZ 200 mg, PB 150 mg

*Localization of seizure onset: LTL, left temporal lobe epilepsy; RTL, right temporal lobe epilepsy*.

*Type of seizure: CP, complex partial*.

*Drugs: CBZ, carbamazepine; CLB, clobazam; CLZ, Clonazepam; OCBZ, oxcarbazepine; PB, phenobarbital; RIS, risperidone; VPA, valproic acid*.

For some of our analyses, we also used ECG data from the PhysioNet Massachusetts Institute of Technology (MIT)-Beth Israel Hospital (BIH) Long-Term Database (1 female; 5 male; age 64.5 ± 16.7 years) (Goldberger et al., 2000).

The study was approved by the Ethics Committee of the Federal University of Santa Catarina, and informed consent was obtained from all the patients.

### 2.2. Methodological framework

**Figure 1:**
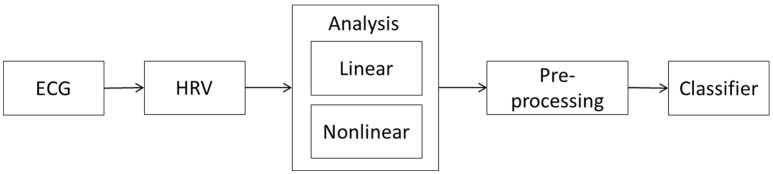
Proposed framework for early seizure detection. The methodology comprises five stages: the acquisition of an ECG recording from the patient, the HRV signal extraction from the recording, the linear and non-linear analysis of the HRV time series, the pre-processing of the parameters derived from the HRV analysis, and the use of a classifier algorithm to identify any preictal states.

Our proposed methodology for the early detection of seizures is based on the analysis of dynamic changes in HRV parameters and the detection of differences between the interictal and preictal periods. Our approach is based on a framework involving five stages, as presented in Figure 1. The next sections present the details of this framework.

#### 2.2.1. ECG data recording

The ECG recording is the first stage of the methodology. HRV time series were extracted from these recordings. It was necessary to collect data in two periods: (1) immediately before seizure onset (up to 10 min before seizure onset), which was defined as the preictal period, and (2) the resting period in between seizures (at least 1 h before or after a seizure), which was defined as the interictal period.

The first indication of the onset of an epileptic seizure may be detected in different ways. For the purposes of this study, we were keen to identify the earliest seizure-related changes in behavior, perception, consciousness or EEG and the moment of transition from the interictal to the ictal phase as precisely as possible. Bearing in mind that the first observable seizure manifestation could be a subjective symptom only reportable by the patient, a change in patient's visible behavior without prior subjective “warning” or a change in the EEG without preceding subjective or visible seizure manifestations, we defined the moment of seizure onset as the earliest of three time-points: (1) when the patient pressed a button to indicate that they had begun to experience seizure symptoms (the “button press” refers to patient's pressing the seizure alarm button synchronized with the video-EEG recording software. A button press will insert a marker in the EEG recording enabling health care professionals to identify the exact timing of onset of a seizure warning or aura). In those cases in which a subjective symptom was the first seizure manifestation this would represent the “clinical onset” of a seizure; (2) at the onset of the first seizure-related changes in the patient's behavior on video (if there is no initial subjective seizure warning and the seizure alarm button has not been pressed, the first visible change in a patient's behavior captured on video or audio during the video-EEG recording marks the clinical onset of a seizure; or (3) at the onset of the first seizure-related changes in the EEG recording (in this case the first occurrence of an ictal EEG pattern, i.e., a change to an EEG pattern typically associated with an epileptic seizure and recognizable by an expert neurophysiologist marks the “EEG onset” of a seizure).

We collected 34 data segments (10 min per segment) capturing preictal periods; 47 data segments totaling 7.8 h (10 min per segment) capturing interictal periods, for training; and 47.4 h of HRV recordings of interictal periods, for testing. We also used a total of 123.6 h of ECG recordings from six healthy subjects to explore the specificity of false positive seizure detections in patients with epilepsy.

#### 2.2.2. HRV signal extraction

The HRV signal extraction is the second stage of the methodology, as described in Figure 1. The approach is based on the HRV time series. Several parameters are extracted from this series, processed, and inputted into the classifier. These signals reflect the activity of the ANS, which in turn is affected by the brain and any seizure related changes in the central nervous system.

The first step in the extraction of the HRV signal was to identify all R-peaks in each ECG recording. To this end, all ECG segments were inspected visually to ensure that the whole sample was artifact-free. The sampling rate used to record the ECG was identified (256 or 512 Hz). Based on this sampling rate, an ECG text file was converted into a corresponding time series, which was fed into custom software using the SciPy: Open Source Scientific Tools for Python (Millman and Aivazis, 2011). Starting with the raw signal, a set of filters was applied to remove low frequency baseline wander and high frequency artifacts, such as muscular activity and power line interference (a high-pass and a low-pass butterworth filters with order 2). Having completed this step, a wavelet-based QRS detection algorithm (Kohler et al., 2002) based on triggering of the R wave detected and computed all consecutive RR intervals values.

In order to eliminate artifacts and ectopic heartbeats, we used a custom algorithm designed to detect all HRV points exceeding 3 times the standard deviation of the sample and changing by more than 30% compared to the previous HRV point. Once identified, these points were removed and a cubic spline interpolation correction method was used to fill the gap in the data thus created. As corrections methods can change the reliability of the HRV assessment (Peltola, 2012), only ECG recordings with <2% ectopic or R waves misdetection instances were included in the analysis.

The proposed methodology is based on the dynamic analysis of HRV parameters. HRV parameters were extracted during multiple periods in each ECG recording (i.e., using an overlapping sliding observation window). For this purpose, two variables were defined: the observation window (*W*_*o*_, the length of the ECG segment used to extract the HRV parameters, in seconds) and the step (*S*, representing the step, in seconds, that the observation window slides through between the start of one extraction period and the start of the next). Therefore, there is a different HRV time series for each extraction period. To define both variables, the characteristics of the signal to be analyzed must be considered. For instance, to analyze low-frequency components, the size of *W*_*o*_ must be large enough to capture the desired frequency band. Thus, for each HRV parameter, a specific *W*_*o*_ was defined.

#### 2.2.3. HRV analysis

The HRV analysis is the third stage of the methodology. The main purpose of this stage is to construct a matrix, called a prediction matrix (*X*), representing data to be processed and inputted into a classifier. To produce the prediction matrix, another variable, the prediction window (*W*_*p*_), must be defined. This is the duration of the period analyzed by the classifier. Thus, a prediction matrix is produced from samples of HRV parameters calculated from the HRV times series (extracted in the *W*_*o*_ range as described earlier) for each step in the ECG recording. Each column of *X* contains the set of HRV parameters of the HRV time series extracted during a specific period. The total number of columns is defined by *W*_*p*_ and *S*.

**Figure 2:**
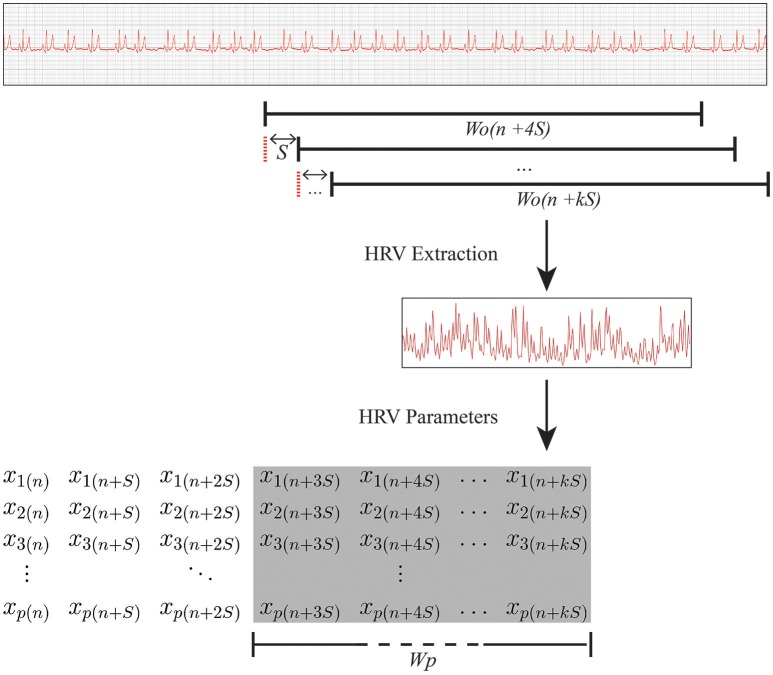
Relationship between *W*_*o*_ and *W*_*p*_. RR interval data were extracted from multiple overlapping observation windows of *W*_*o*_ seconds in the ECG recording. Subsequently, a set of HRV parameters was calculated and inserted into prediction matrix *X* (which is shown in the shaded area of the overall matrix), with duration *W*_*p*_ (i.e., the duration of the period analyzed by the classifier). *k*, the last step of the overlapping observation window slide in a ECG sample; *S*, step, in seconds, that is the overlapping observation window slides through between the current extraction period and the start of the next one; *W*_*o*_, observation window, in seconds, the length of the ECG segment used to extract the HRV parameters; *W*_*p*_, prediction window, in seconds, the duration of the period analyzed by the classifier; *x*_*p*_(*n*), the *p*^*th*^ parameter extracted in the *n*^*th*^ sample.

A prediction matrix is defined as follows:

(1) $$X=\left[\begin{array}{ccc} x_{11} & \cdots & x_{1n}\\ \vdots & \ddots & \vdots \\ x_{p1} & \cdots & x_{pn} \end{array}\right]\in {ℜ}^{p\times n},$$

where *p* is the number of HRV parameters and *n* the number of samples, with $n=\frac{W_{p}}{S}$. The HRV parameters are in turn calculated from the HRV signal extracted in the *W*_*o*_ range, as shown in Figure 2.

##### 2.2.3.1. HRV parameters

Over the last decade, researchers have proposed several analyses and measures to evaluate HRV (Lotufo et al., 2012; Kranjec et al., 2014). The methods used in our study were based on linear and non-linear analyses in the time and frequency domains.

Time domain measures include basic parameters, such as the mean RR interval. The parameters in the time domain used in this study were as follows:

SDNN (ms): The standard deviation of all normal RR intervals (SDNN). This parameter provides information on all components that contribute to the HRV, and it is very dependent on the total time used for the HRV analysis. In patients with epilepsy, the SDNN has a lower value than in healthy subjects (Ponnusamy et al., 2011, 2012; Lotufo et al., 2012), which is related to the reduced parasympathetic activity in those with epilepsy.

RMSSD (ms): The root mean square of the sum of the squared differences of successive normal RR intervals (RMSSD). This parameter reflects parasympathetic activity (DeGiorgio et al., 2010), which is expected to be reduced in patients with epilepsy.

The HRV signal is composed of multiple frequencies that provide information about sympathetic and parasympathetic activity. The contributions of different frequencies to the total RR variability are usually separated using a fast Fourier transform (FFT)-based power spectral density (PSD) analysis. The two main spectral bands that comprised the signal's spectrum used in this work are as follows:

LF: The PSD of the low-frequency range with components ranging from 0.04 to 0.15 Hz. The LF component is largely related to sympathetic activity but can also be modified by vagal activity (Ponnusamy et al., 2012). Most authors consider LF a measure of sympathetic activity (Clifford, 2002).

HF: The PSD of the high-frequency range with components ranging from 0.15 to 0.4 Hz. The HF component is considered a measure of parasympathetic activity (Clifford, 2002).

The HRV signal has a non-linear nature and the use of linear techniques may not allow the identification of abnormal HRV patterns. Therefore, non-linear methods have increasingly been used for HRV signal analyses. In this study, we used the following two non-linear parameters:

SampEn: The sample entropy (SampEn) is an entropy parameter (Richman and Moorman, 2000), the calculation of which allows the quantification of the regularity and complexity of time series. In patients with epilepsy, marked differences in entropy have been observed between ictal and interictal periods (Ponnusamy et al., 2011, 2012). Entropy decreases during the ictal phase probably associated with increased sympathetic activity.

Lorenz plot: The Lorenz plot is a non-linear dynamic technique that can be used to indicate fluctuations in RR interval time series. This method involves plotting each *RR*(*n*) interval against the subsequent interval, *RR*(*n* + 1). In the resulting chart, the length of the transverse axis (*T*) reflects the variability of the heart rate, which is related to the dominance of the parasympathetic system. The length of the longitudinal axis (*L*) reflects the general behavior of the HRV signal due to the influences of both systems, sympathetic and parasympathetic. The measures derived from this plot are as follows:

CSI: The cardiosympathetic index (CSI) is calculated as follows:
  (2) $$CSI=\frac{L}{T}.$$
CVI: The cardiovagal index (CVI) is calculated as follows:
  (3) CVI=log[L×T].

It has been suggested that these indices provide complementary information about parasympathetic and sympathetic contributions to HRV parameters based on spectral analysis alone (Toichi et al., 1998).

Lorenz plots have been widely used in HRV analyses of different disease groups (Toichi et al., 1998; Ponnusamy et al., 2011, 2012). In patients with epilepsy, a reduction in variability is observed in the ictal period, both qualitatively by observing the Lorenz plot and quantitatively, as reflected by higher CSI values. More recently, this method has also been used for the detection of epileptic seizures (Jeppesen et al., 2015).

In summary, we used seven HRV metrics (SDNN, RMSSD, LF, HF, SampEn, CSI, and CVI) to construct a matrix *X* ∈ *R*^*P*×*T*^ for each recording analyzed.

#### 2.2.4. Pre-processing

The pre-processing is the fourth stage of the methodology. In this step of the analytic process, we calculated the covariance matrix of *X* followed by the eigendecomposition to be inputted into the classifier. The main aim of this process was to obtain a vector with information on the principal components at each moment in time.

A covariance matrix is a square matrix that contains the variance and covariance associated with several variables. These are important descriptors that indicate the dispersion of a distribution. Consider the case in which we have *p* parameters from *n* samples. We define the covariance matrix *R*_*xx*_ to be a symmetric *p* × *p* matrix with element *r*_*ij*_ equal to the covariance between variable *i* and variable *j*. Naturally, the *i*_*th*_ diagonal element of this matrix contains the covariance of variable *i* with itself, i.e., its variance. Accordingly, the covariance matrix of *X* can be obtained as follows:

(4) $$R=\frac{1}{k-1}\sum_{i=1}^{k}\left(X_{i}-\bar{X}\right){\left(X_{i}-\bar{X}\right)}^{\prime }$$

where *k* = *p*. Since we have assumed that there are *p* parameters, the rank of matrix *R* is necessarily *p*, as long as the parameters are not correlated. Therefore, the eigendecomposition of matrix *R* provides *p* non-zero eigenvalues and *p* eigenvectors.

To define good parameters as inputs for the classifier, we selected the principal components of *X* once it was in an arrangement that best represented the distribution of the data. Thus, the vector inputted into the classifier was composed of the largest eigenvalue (λ) plus the eigenvector (*v*) of the largest eigenvalue representing the maximum variance of the data. The vector was defined as follows:

(5) $$\Lambda =[\lambda ,v_{1},\ldots ,v_{p-1}]\in {ℜ}^{p}$$

where *p* is the number of parameter types. During these calculations and for the classification process, neither the signals nor the eigenvalues were normalized but the eigenvector components were.

#### 2.2.5. Classifier

The use of a classifier is the last stage of the methodology. According to the scheme shown in Figure 1, in this stage, the input vector (which was calculated in the pre-processing stage) is classified according to whether it is associated with a preictal or interictal state. We propose the use of supervised machine learning algorithms for the early detection of seizures based on changes in the eigenvalues and eigenvectors of the HRV parameter covariance matrix.

SVM classifiers have been widely used in recent research as a machine learning solution for a highly diverse range of problems. Applications in the field of medicine have yielded several notable results using ECG recordings (i.e., results regarding ECG quality estimation and computer-aided morphological analysis) (Jankowski et al., 2003; Morgado et al., 2015). The pattern recognition algorithm deals with a convex optimization problem involving a maximum margin hyperplane separating two classes. This hyperplane depends on a subset of training patterns called support vectors (Schölkopf and Smola, 2002).

Using a similarity function, it is possible to map the data onto a higher-dimensional space and then apply the hyperplane strategy. For this application, the Gaussian kernel was employed, as defined by the following equation:

(6) $$k(u,v)=exp(-\frac{{\left\|u-v\right\|}^{2}}{\gamma }),$$

where **u** is the input sample, **v** is the landmark defined by the training set, and ||.|| represents the Euclidean distance operator (Schölkopf and Smola, 2002). Thus, the main aim was focused on the standard classification problem that consists of constructing a classifier to distinguish between two disjointed sets of points in a Euclidean space.

For this purpose, two important parameters must be considered. The penalty parameter (*C*), which controls model overfitting, and the parameter gamma (γ), which controls the model's degree of nonlinearity. To obtain the optimum classification performance, it was necessary to find the best combination of these parameters based on training accuracy as described in the next section.

A major advantage of using SVM for distinguishing between interictal and preictal periods is its robustness regarding different types of interictal patterns.

### 2.3. Training process

The training process involved structuring a model that had received parameter matrices from preictal and interictal periods. To construct, train, and validate the model, three steps were necessary: (1) constructing all the prediction matrices; (2) defining the training setup, and (3) evaluating the performance.

#### 2.3.1. Prediction matrix construction

To construct the prediction matrices for training the classifier, we created an algorithm that extracts HRV features from the prediction window. The algorithm was defined as follows:

**Algorithm 1:** Prediction matrix construction

1: *t* = *actualTime* − *W*_*o*_ − *W*_*p*_
2: **while** *actualTime* ≥ *t* **do**
3: window ← data from t to *t* + *S*
4: Extract RR intervals from window
5: Compute HRV parameters
6: Fill prediction matrix with a column of p parameters
7: *t* ← *t* +*S*
8: **end while**

Importantly, the *actualTime* variable was defined as an instant in which we know an event of interest is occurring or about to occur, i.e., interictal or preictal periods in this case. Therefore, we applied a causal signal analysis (Allen and Mills, 2004) with the point of interest based on *actualTime* (i.e, the output was always computed from present and past inputs).

In this process, the observation window acts as a circular buffer, meaning that for each step *S* the window removes data from the oldest *S* seconds and adds data from the next *S* seconds.

#### 2.3.2. Training setup

Due to the limited availability of data (i.e., only data on 34 seizures were used), we used a leave-one-out cross-validation (LOOCV) approach to derive a more accurate estimate of model prediction performance.

Training data were generated and separated as follows:

1. We initially selected 33 samples of preictal data for training and one sample of preictal data for test purposes. The training process was repeated 34 times as part of the cross-validation process.
2. Interictal training samples were generated using randomly chosen intervals from the periods when no seizure had occurred 1 h before or after the sample period. A total of 47 interictal HRV matrices were extracted to make up the training set, totaling 7.8 h of data. The training process also was repeated 47 times as part of the cross-validation process.

We calculated a set of prediction matrices based on the recordings before seizure onset in order to train the classifier to categorize the input as being associated with the preictal period. These matrices were constructed based on a *W*_*p*_ = 60 s and *S* = 10 s, representing 6 columns of HRV parameters that preceded the moment of interest (i.e., seizure onset). For each value in each column, *W*_*o*_ seconds of HRV data were selected to extract the HRV parameters in order to construct the matrix. A *W*_*o*_ value was defined for each parameter, as shown in Table 2. Thus, each prediction matrix represents a moment in the signal involving causal data.

**Table 2:** *W*_*o*_ values for each HRV parameter.

**HRV parameter**	**SDNN**	**RMSSD**	**LF**	**HF**	**SampEn**	**CSI**	**CVI**
*W*_*o*_ value (s)	60	60	180	180	60	60	60

*CVI, cardiovagal index; CSI, cardiosympathetic index; HF, high frequency; LF, low frequency; RMSSD, root mean square of the sum of the squared differences of successive normal RR intervals; SampEn, sample entropy; SDNN, standard deviation of all normal RR intervals*.

The dimension of these matrices was 7 × 6, as defined in Equation (1). Each *X* was used to calculate a vector (Λ), which was used in the early detection and prediction model.

To define the best combination of SVM parameters (*C* and γ), each combination was checked using the cross-validation approach and the parameters with the best cross-validation accuracy were selected.

#### 2.3.3. Evaluation of early detection performance

The most commonly used performance measures to evaluate an early detection or prediction model are sensitivity and false positive rate. In seizure prediction studies, a seizure is considered to have been predicted correctly if there is at least one warning within the preceding prediction horizon (Wang et al., 2013). In this study, the sensitivity was calculated based on the prediction of seizures from 5 min before to just before seizure onset. This prediction horizon was defined based on the number of true positives in each minute before seizure onset; the true positive rate decreased significantly for the time periods >5 min before seizure onset. Furthermore, given the nature of the causal signal analysis, the sum of *W*_*o*_, *W*_*p*_ and prediction horizon period can not be higher than the preictal total period in order to provide sufficient ECG data for analysis.

The LOOCV approach was used to calculate the sensitivity. Each false positive rate was calculated by applying the model to the relevant set of samples (based on the ECG recordings from the interictal periods and healthy subjects). Given previous results based on the use of EEG and ECG recordings (Park et al., 2011; Fujiwara et al., 2016), we defined the acceptable false positive rate as a rate of <0.5 h^−1^.

## 3. Results

To assess early epileptic seizure recognition, we used (1) a set of ECG signals recorded during (and immediately before) at least one seizure, (2) a set of ECG signals recorded during interictal periods, with a total length of 55.2 h (7.8 h for training and 47.4 h for testing), and (3) a set of ECG signals from the MIT-BIH Long-Term Database with a total length of 123.6 h (Goldberger et al., 2000).

For each assessment of the methodology, a set of HRV parameters was calculated using the set of *W*_*o*_ values shown in Table 2, and *S* = 10 s, which were determined on the basis of the particular characteristics of the different HRV parameters. Sympathetic and parasympathetic tone can vary considerably over a 24-h cycle and can change abruptly in response to external and internal stimuli, such as fear and pain. Therefore, we used relatively short observation windows (see Table 2) to minimize the effects of ANS responses to external influences that could have interfered with the analysis of interest.

**Figure 3:**
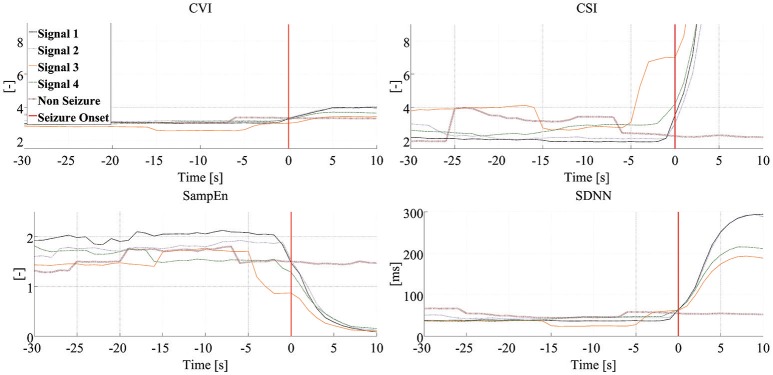
Dynamics of HRV parameters. Each panel presents dynamic changes in one particular HRV parameter during four seizures and one interictal period experienced by the same patient. Clinical/EEG seizure onset is represented by the vertical red lines. Each of the signals associated with seizure-related findings has been synchronized to seizure onset. CSI, cardiosympathetic index; CVI, cardiovagal index; SampEn, sample entropy; SDNN, standard deviation of all normal RR intervals.

**Figure 4:**
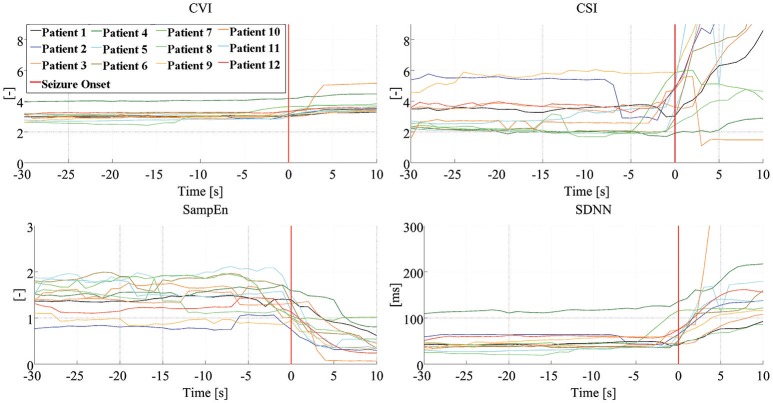
Dynamics of HRV parameters. Dynamic changes of four HRV parameters, from 12 patients, with the signals synchronized to seizure onset. Clinical/EEG seizure onset is represented by the vertical red lines. CVI, cardiovagal index; CSI, cardiosympathetic index; SampEn, sample entropy; SDNN, standard deviation of all normal RR intervals.

Figure 3 shows the dynamics of four HRV parameters (*CVI, CSI, SampEn*, and *SDNN*) in five signals from one patient. Four of these signals were associated with seizure onset (and have been synchronized to the point of seizure onset shown in the figure) while one shows the signal during an interictal period. Note that different HRV parameters show similar dynamic changes around the time of seizure onset while the signals in the interictal periods do not show any similar changes. Figure 4 shows the dynamics of the four HRV parameters from 12 patients. Although there were differences in patient characteristics, the dynamic behavior of the HRV parameters was similar. These stereotypical dynamic changes in HRV parameters around the time of seizure onset suggest that the parameters could be useful for the early detection or prediction of epileptic seizures.

**Figure 5:**
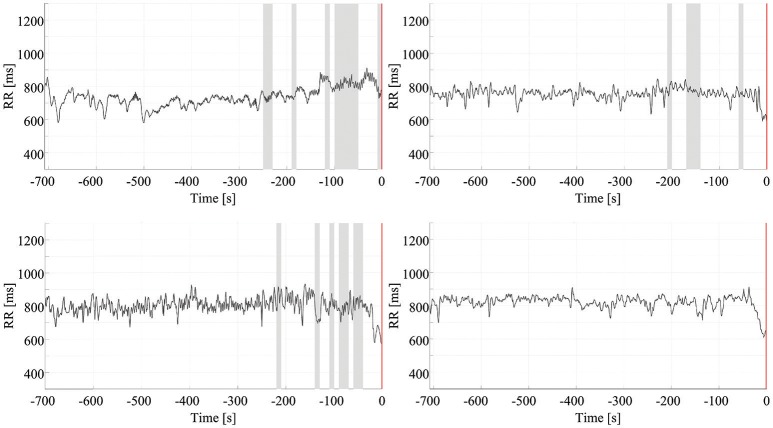
Early detection of seizures. Each chart presents the results of the early detection/prediction model, with positive output (indicating seizure prediction) highlighted using shading. Clinical/EEG seizure onset is represented by the vertical red lines. The top two panels show successful early prediction/detection of seizures in two different patients. The bottom panels are from a third patient: the left panel shows a successful seizure detection the right panel an example of a seizure which was not detected.

The optimum combination of SVM parameters, *C* = 4.3 and γ = 0.5, reached an accuracy of 95.6%. Thus, the results for the prediction of impending seizures from 5 min before to just before clinical/EEG seizure onset indicated a sensitivity of 94.1%, based on a detection rate (up to 5 min before onset) of 32 of the 34 seizures. Figure 5 presents the prediction results for four seizures. The upper two panels show the prediction of seizures of different patients, and the lower two panels are based on recordings from a third patient. These two panels show that, in one case, accurate seizure prediction was not achieved.

**Figure 6:**
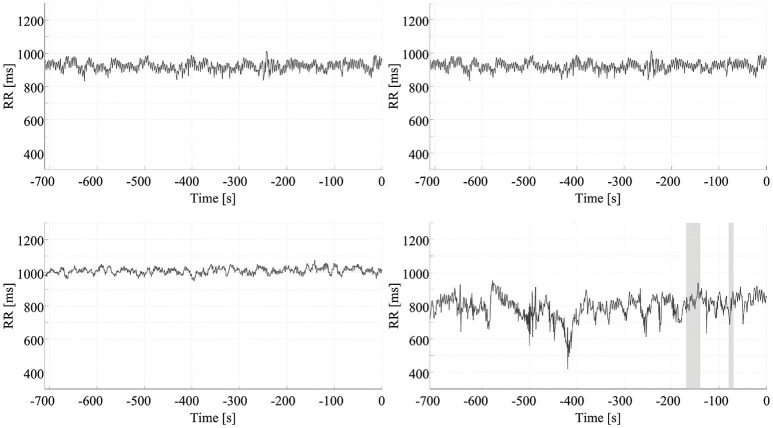
False positive rate of seizure prediction. Each chart presents the results of the early detection/prediction model, with positive output (indicating seizure prediction) highlighted using shading. Each of the graphs show the HRV signal from a different ECG recording of an interictal period.

The false positive rate in the interictal ECG segments was 0.49 false positives (FP)/h. For the data from the MIT-BIH Long-Term Database, the false positive rate was 0.19 FP/h. Figure 6 shows four results used to calculate the false positive rate for interictal periods. All results are summarized in Table 3.

**Table 3:** Summary of results.

**Sensitivity**	**Accuracy**	**FP/h^a^**	**FP/h^b^**
94.1%	95.6%	0.49	0.19

a *False positive rate in the interictal ECG segments*.

b *False positive rate in ECG segments from MIT-BIH Long-Term Database*.

## 4. Discussion

In this study, we propose a methodology for the early detection and prediction of epileptic seizures using HRV signals only. This method is based on the extraction of a range of HRV parameters that reflect changes in sympathetic and parasympathetic tone around the onset of epileptic seizures. The methodology could be used in closed-loop seizure treatment systems or seizure warning devices. This paper presents a classification approach that combines a linear signal subspace analysis (i.e., the eigendecomposition of covariance matrices) with an interpretable machine learning process. The feasibility of implementing the methodology was investigated by first analyzing different timepoints prior to seizure onset (to assess the maximum prediction time that can be achieved with this methodological configuration) and also by analyzing ECG data on healthy subjects.

Epilepsy and seizures can have profound effects on both subdivisions of the ANS, the sympathetic and parasympathetic systems. ANS is fundamental to homeostasis and including regulation of heart rate. The parasympathetic output (mediated by the vagus nerve) and sympathetic output (controlled by neurons in the rostral medulla) to the heart are modulated by the central autonomic network. This includes the insular cortex, orbitofrontal cortex, cingulate, amygdala, hypothalamus and peri-aqueductal gray matter. The insula and prefrontal cortex are considered as the key representations of the autonomic nervous system at the cortical level. Epileptic discharges affecting these parts of the brain affect the functioning of these components of the central autonomic network.

Heart rate variability can be used as a tool to provide information about the functional state of the autonomic nervous system. Ictal activation of the ANS can be detected by analyzing HRV on electrocardiography (Sevcencu and Struijk, 2010) as HRV is a mirror of neuronal influences on the cardiac pacemaker. HRV parameters reflect the beat-to-beat variability of the intrinsic oscillators, which are controlled by the sympathovagal balance. In this sense, heart rate variability can be considered as a biomarker of the autonomic dysfunction caused by seizures. As such the early recognition of such HRV changes could be clinically useful for the rapid detection and, perhaps, prediction of impending seizures.

The methodology is based on the observation that HRV parameters differ significantly between interictal, preictal, and ictal phases. However, to observe the dynamics of these variations and analyze the preictal changes in the parameters, the HRV parameters must be calculated continuously. The choice of the observation and prediction windows is of great importance to the success of early seizure detection or prediction algorithms. In addition, further study is needed on the influence of physiological activities that potentially affect HRV parameters, signal acquisition quality, ectopic heartbeats, seizure type, seizure focus location, and the patient's age before the analysis of HRV parameters can be used in clinical settings for seizure detection or prediction. This research will require larger prospective studies.

Having said that, the relative ease of ECG acquisition from patients means that there is considerable clinical interest in ECG-based seizure detection and warning systems (Osorio and Manly, 2014). Furthermore, these systems could be made more effective by using a multimodal monitoring approach that also incorporates other biological or behavioral signals. ECG-based algorithms are certainly more practical and convenient than devices using EEG signals. The high signal-to-noise ratio and ease of recording favor ECG over EEG-based approaches. In addition, as about 82% of epileptic seizures are associated with ictal tachycardia (which often precede ictal EEG changes) (Eggleston et al., 2014), our proposed method of seizure detection/prediction based on HRV parameters may be very useful for seizure warning devices.

Approaches for early detection of seizures based on HRV parameter dynamics may also be useful for treatment. Vagal nerve stimulation devices provide safe and effective treatment for refractory epilepsy. Vagal nerve stimulation is usually delivered intermittently in an open-loop fashion. Recently, an automated seizure detection system that uses ictal tachycardia to trigger vagal nerve stimulation in a closed-loop fashion was piloted and found to be potentially efficacious for the management of difficult-to-treat patients with epilepsy (Boon et al., 2015). Our methodological approach of HRV-derived seizure detection/prediction may be used in a similar fashion in closed-loop seizure management devices. It may prove to be a more responsive and specific approach than tachycardia-based approaches.

The use of HRV parameters for the early detection and/or forecasting of epileptic seizures may facilitate the development of new devices for continuous monitoring in other applications that involve time-variant biological signals and applications associated with closed-loop electroceutical devices. Furthermore, early detection of epileptic seizures can play a key role in enabling patients to reach a specialized treatment center promptly and in optimizing the diagnosis and treatment of epilepsy. Overall, these factors may be linked to a reduction in mortality rates and comorbidities (Maguire et al., 2016; Granbichler et al., 2017).

Despite the high computational cost, we used the LOOCV approach due to the possibility of training the classifier using the greatest amount of data in each case, which increases the probability of producing an accurate classifier (Witten et al., 2011). However, the high variance associated with this approach can lead to unreliable estimates. Nevertheless, we evaluated the false positive rate in an independent dataset and, furthermore, 32 of the 34 outputs (considering only preictal periods) were true positive outputs, indicating that the model is reliable.

Future research will need to include a greater number of patients, in order to examine the possible effects of clinical characteristics, such as the patient's age, seizure type, and, in particular, HRV parameters. Future studies using the methodology presented in this study should address the limitations of the present study, which include the small case number, the fact that only focal seizures were studied, and the moderate quantity of interictal ECG recordings analyzed. There was a difference in the gender ratios of the patient and control groups in this study. Although there are indications of gender differences between some HRV parameters (Koenig and Thayer, 2016), these differences have not been replicated in studies of patients with epilepsy (Lotufo et al., 2012). What is more, the most important analyses in the present study rely on comparisons of ictal and interictal data from the same patient. Nevertheless, it would be important for confirmatory future studies to ensure that the observations made here are not affected by patient's gender. Furthermore, new HRV-based approaches, such as non-linear analyses based on chaos theory could be integrated into the methodology to explore its full potential for assessing ANS dynamics in multiple physiological conditions.

## 5. Conclusion

It is feasible to use the dynamics of HRV parameters for the early detection and, potentially, the prediction of epileptic seizures. The use of SVM for classifying input according to whether it is associated with an interictal or preictal period is a robust technique regarding the classification of different types of interictal patterns. We achieved an acceptable (<0.5 FP/h) false positive rate of 0.49 FP/h for ECG recordings from all the interictal periods, with a sensitivity of 94.1% for seizure prediction based on recordings capturing the period 0–5 min before seizure onset. For the recordings from the MIT-BIH Long-Term Database, the false positive rate was 0.19. The results of this study show that early detection of epileptic seizure is possible using HRV parameters.

## Ethics statement

This study was carried out in accordance with the recommendations of Ethics Committee of the Federal University of Santa Catarina with written informed consent from all subjects. All subjects gave written informed consent in accordance with the Declaration of Helsinki. The protocol was approved by the Ethics Committee of the Federal University of Santa Catarina.

## Author contributions

JP, RH, BN, RW, MR, AS, AP, and JM participated in the design of the entire study and helped to draft the manuscript. JP and RH contributed to the model implementation and simulation and interpreted the data. All the authors read and approved the final manuscript.

### Conflict of interest statement

The authors declare that the research was conducted in the absence of any commercial or financial relationships that could be construed as a potential conflict of interest.
